# Morphological and Genome-Wide Evidence of Homoploid Hybridisation in *Urospermum* (Asteraceae)

**DOI:** 10.3390/plants11020182

**Published:** 2022-01-11

**Authors:** Jaume Pellicer, Manica Balant, Pol Fernández, Roi Rodríguez González, Oriane Hidalgo

**Affiliations:** 1 Institut Botànic de Barcelona (IBB, CSIC-Ajuntament de Barcelona), Passeig del Migdia s.n., Parc de Montjuïc, 08038 Barcelona, Spain; manica.balant@ibb.csic.es (M.B.); pol.fernandez@csic.es (P.F.); roirgonzalez@ibb.csic.es (R.R.G.); 2 Royal Botanic Gardens, Kew, Kew Green, Richmond TW9 3AE, UK

**Keywords:** chromosomes, genome size, homoploid hybrid, Mediterranean region, phenotype, repetitive DNA

## Abstract

The genus *Urospermum* is distributed in the Mediterranean region and Macaronesia, and has been introduced to other extra-Mediterranean regions. Although the two species constituting the genus, *U. dalechampii* and *U. picroides*, are frequently found together, hybrids have so far only been reported once, from Morocco. However, we found certain individuals in Catalonia, whose intermediate morphology suggested a potential hybrid origin. In this study, we applied morphological and molecular methods to investigate the origin of those individuals. Intermediate features at phenotype, karyological, cytogenetic, and genomic levels were identified in morphologically intermediate individuals, supporting their homoploid hybrid origin. Chloroplast sequence data suggest that *U. dalechampii* is the maternal progenitor of the hybrid. Together with the intermediate traits displayed, the lack of fertile seeds suggests that hybrids are probably F1. Future monitoring studies will be, nonetheless, needed to evaluate the extent of hybridisation and its potential impact on the biology of the genus.

## 1. Introduction

The genus *Urospermum* Scop. (Asteraceae, Cichorieae) is distributed across the Mediterranean region and Macaronesia, and has been introduced to other extra-Mediterranean regions [1]. This small genus is made up by two species, *U. dalechampii* (L.) Scop. ex. F.W.Schmidt and *U. picroides* Scop. ex. F.W.Schmidt, which diverged during a late Miocene to Quaternary timeframe [2]. Morphologically, both species present capitula with an involucre of usually eight bracts of equal length arranged in a row, and connate in their lower third, which makes the genus easily recognisable in the field. Both species are, however, well differentiated, in particular by the indumentum of involucral bracts (and the overall plant) that is softly hairy in *U. dalechampii*, while hispid and spinulose in *U. picroides*. Flowers are pale yellow in *U. dalechampii* and intense yellow in *U. picroides*. The two species also differ on the basis of capitula size and shape, number of capitula per annual growth, and life cycle [1]. Besides these morphological peculiarities, each species can be also distinguished upon karyological and cytogenetic features. The species *U. picroides* has a chromosome number of 2n = 10, and a genome size reported of 1.38 pg/2C [3]. In contrast, *U. dalechampii* has 2n = 14 chromosomes and a larger genome size (1.95 pg/2C [4]). Although these two species are frequently found together, especially in the South of France and East of Spain, hybrids have so far only been reported once, in populations from Morocco [5].

In spring 2021, we found some individuals in Catalonia whose intermediate morphology, compared to the above-mentioned species, suggested potential hybridisation events in the area, which we decided to study in further detail. Natural hybridisation is a recurrent and important trait in the evolution of many plant lineages, which can underpin the rise of new species [6]. Indeed, this phenomenon has been widely associated to speciation, given the subsequent continued genomic divergence with respect to the parental genomes [7]. Homoploid hybridisation (with no change of ploidy level involved) has been for long-time considered to be less common than allopolyploid hybridisation, but the actual rates and their impact on speciation continue to be a source of debate [8]. In Asteraceae, it is relatively easy to find examples of homoploid hybrids in several genera, such as for example in *Argyrantheum* Webb [9], *Centaurea* L. [10] or *Helianthus* L. [11], but model systems for understanding allopolyploidy in the family represent the bulk of research in this field (e.g., *Tragopogon* L. [12]). Among many other consequences, hybridisation impacts directly in the genome size of the resulting hybrid offspring. Hybrids can display either a suite of sizes that fall between the nuclear DNA contents of the parental genomes, or even display more heterogeneous patterns depending on the level of polyploidy and subsequent introgression [10,13].

In general, genome scanning techniques are necessary to unravel the extent of hybridisation and polyploidy from an evolutionary perspective [14,15]. However, complementary techniques such as flow cytometry can provide preliminary evidence of these processes through quantification of the nuclear DNA contents. Indeed, those are sometimes necessary to interpret allelic frequencies when complex allopolyploid networks are involved [13,16]. Besides morphological differences, *U. dalechampii* and *U. picroides* have been reported to have different genome sizes, and therefore potential hybrids could display intermediate sizes if homoploid, or additive patterns if hybridisation resulted in polyploid cytotypes or in activation of specific DNA repeats. Certainly, plant genomes are colonised by repetitive DNA, which consists of two main categories, namely dispersed mobile elements and tandem repeats [17]. DNA transposons and retrotransposons are commonly known as transposable elements, given their ability to move across the genome, being long terminal repeat (LTR) retrotransposons dominant across the genomic landscape in plants [18,19]. The evolutionary dynamics of repetitive elements in the genome can be influenced by hybridisation or polyploidy, among others [20,21,22], which can act solely or in concert [23], thus influencing changes in repeat composition due to changes in rates of amplification and deletion. The extent at which these opposite forces act, are key drivers of genome size evolution. Next generation sequencing techniques have seen an unprecedented development in the last decades, enabling comprehensive understanding of plant genomes beyond model plants. Short-read genome skimming products can be used to feed clustering pipelines, which reconstruct and assemble repetitive elements based on all-to-all similarity comparisons, without the need of a reference genome (e.g., RepeatExplorer, [24]), and have become frequently-used to analyse repetitive DNA dynamics and genome evolution in plants [22,25,26,27].

Bearing all the above in mind, in this study we gather and combine morphological, cytogenetic and genomic data in *Urospermum* species and the suspected putative hybrids. Specifically, we aim to (i) evaluate whether changes in phenotypes across specimens can be associated to hybridisation. (ii) To characterise chromosomally and through genome size both the parental species and the suspected hybrids, and thus confirm if homoploid or polyploid hybridisation occurs in the area of study. And (iii), to characterise the repetitive genome of these taxa and evaluate the impact of hybridisation in the organisation of repetitive elements in hybrid offspring.

## 2. Results

### 2.1. Morphological Characterisation of the Genus Urospermum

**Figure 1:**
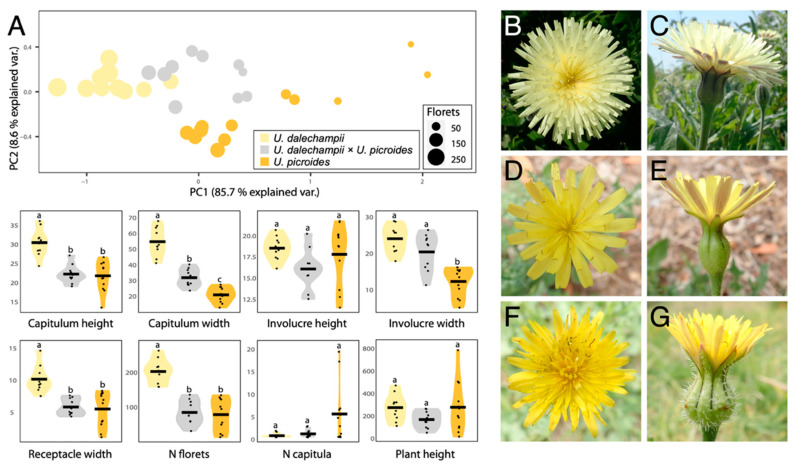
(**A**) PCA and violin plots showing morphological trait distribution in *Urospermum dalechampii* (yellow), *U. dalechampii* × *U. picroides* (grey) and *U. picroides* (orange). PCA of log-transformed capitula measurements was performed on a dataset including capitulum height, capitulum width, involucre height, involucre width and receptacle width. Horizontal lines of violin plots represent mean values for the variables, and dots represent values for each individual. Letters above violins indicate which groups are statistically different from one another. (**B**–**G**) Capitula’s frontal and lateral views of *U. dalechampii* (**B**,**C**), *U. dalechampii* × *U. picroides* (**D**,**E**), and *U. picroides* (**F**,**G**).

Results of the morphological characterisation are presented in Figure 1 and Table 1. Together, PC1 and PC2 capture 94.3% of the variation in the data. In both principal components, the receptacle width is the most important trait in differentiating between taxa, followed by the capitulum width (Figure 1A). Hybrids occupy an intermediate morphospace region, but slightly overlap that of *U. dalechampii*. Notwithstanding, when considering the characters separately, they most often show measurements closer to *U. picroides* than to *U. dalechampii* (e.g., capitulum height, receptacle width and floret number). This could suggest that hybrids, although morphologically intermediate to parental taxa, are somewhat more similar in shape to *U. dalechampii*, but closer in size to *U. picroides*.

### 2.2. Chromosome Counts

**Figure 2:**
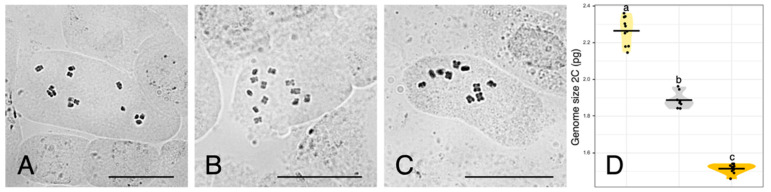
(**A**–**C**) Somatic metaphase plates of *Urospermum dalechampii*, 2n = 14 (**A**), *U. dalechampii* × *U. picroides*, 2n = 12 (**B**), and *U. picroides*, 2n = 10 (**C**). Scale bars = 10 μm. (**D**) Violin plots showing the distribution of genome size in *U. dalechampii* (yellow), *U. dalechampii* × *U. picroides* (grey) and *U. picroides* (orange). Horizontal lines of violin plots represent mean values for the variables, and dots represent values for each individual. Letters above violins indicate which groups are statistically different from one another.

Chromosome counts are depicted in Figure 2A–C. Reported numbers are as follows: *U. dalechampii* (2n = 14), *U. picroides* (2n = 10), and their putative hybrid *U. dalechampii* × *U. picroides* (2n = 12).

### 2.3. Genome Size Measurements

Flow cytometry results are provided in Figure 2D and Table 2. Both parental species present significantly different genome sizes, *U. dalechampii* having a genome size of 2.26 pg/2C, and *U. picroides* of 1.51 pg/2C. Their putative hybrid has a genome size corresponding to the average value between both (1.89 pg/2C).

### 2.4. Individual Repeat Content and TE Annotation

**Figure 3:**
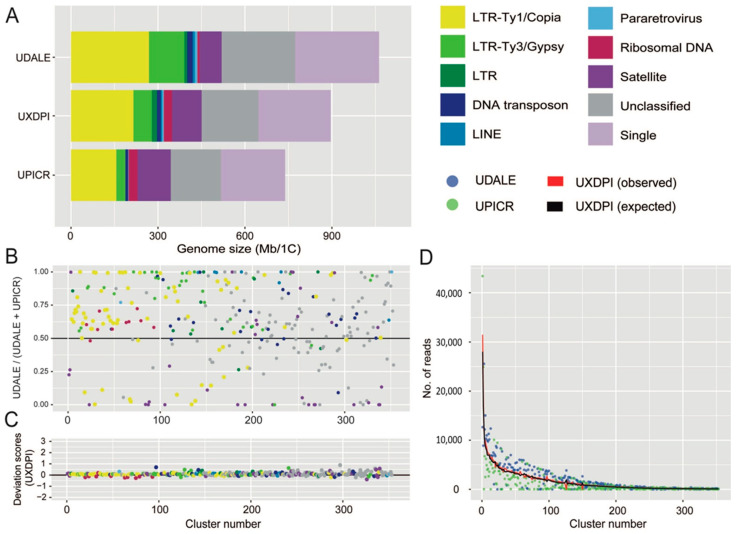
(**A**) Genomic composition of *Urospermum dalechampii* (UDALE), *U. dalechampii* × *U. picroides* (UXDPI) and *U. picroides* (UPICR). Estimates of the genomic abundances (in Mb/1C) of different repeats are colored by repeat class. The size of the unclassified (grey) and low/single copy fraction (pale purple) of each genome is also shown. (**B**) Comparison of cluster abundances in the parental species *U. dalechampii* and *U. picroides*, irrespective of cluster size. Read clusters falling along the black line (i.e., value of 0.5) have the same proportion in both species. Those above the 0.5 are more abundant in *U. dalechampii* and those below this threshold are more abundant in *U. picroides*. (**C**) Deviation scores of observed cluster sizes from the expected values in the putative hybrid (UXDPI). Clusters above and below 0 line represent larger and/or smaller values than the expected values from parents. (**D**) Scatter plot of genomic contributions (read clusters) of *U. dalechampii* (blue dots) and *U. picroides* (green dots) to the hybrid *U. dalechampii* × *U. picroides* (red line) and the expected values calculated assuming a 50% genome dosage (black line).

Detailed information regarding the number of reads analysed for each taxon and their corresponding genomic coverages are given in Table 3. The repetitive fraction of the genomes analysed ranged from 69.744% in *U. picroides* to 74.164% in *U. dalechampii*, the hybrid taxon displaying intermediate value (i.e., 70.793%, Table 4). The annotation and classification of the most abundant clusters is presented in Table 4 (and Supplementary Table S1) and represented in Figure 3A. Small clusters (GP < 0.01%), together with those above this threshold that failed to match the classifications from REXdb were left as unclassified (22.628% in *U. dalechampii*, 23.587% in *U. picroides* and 22.768% in *U. dalechampii* × *U. picroides*, Table 4). The most abundant type of DNA repeats in all three genomes analysed were LTR retroelements, especially those belonging to Ty1/Copia superfamily (Table 4, Figure 3), which comprised c. 20–24% of the genomes studied. Of the eight main lineages recovered, Ty1Copia-SIRE clusters were, by far, the most abundant lineage, with genome proportions around 17–18% (Table 4). Among Ty3/Gypsy lineages, the presence of Tekay elements was much higher than any other lineages across all taxa studied (Table 4). However, whilst in *U. dalechampii* Ty3/Gypsy LTR were found as the second most abundant type of identified repeats, in *U. picroides* and the putative hybrid we detected changes in repeat composition, being satellite DNA much more abundant than Ty3/Gypsy elements, and accounting for meaningful genomic proportions of about 15.638% in *U. picroides* (Ty3/Gypsy = 4.255%).

### 2.5. Comparative Repeat Dynamics in Urospermum and the Putative Hybrid

The comparative analysis including both parental species and the resulting hybrid revealed highly variable abundance composition of shared elements between both parental genomes, even after removing cluster size effect (Figure 3B). Most shared clusters were, indeed, more abundant in *U. dalechampii* than in *U. picroides* irrespective of their classification, except for satellite DNA, which were, in general, more abundant in *U. picroides*. In a recently formed hybrid, a proportional inheritance of DNA repeats from each of the progenitors corresponding to their genome sizes can be expected. Overall, most cluster abundances recovered in the hybrid were intermediate between both parents, and very similar to the expected values assuming proportional genomic contribution by each of the parental donors, with only small deviations being detected (Figure 3C,D). Higher levels of variation were observed in smaller clusters, which had minor impact on the overall genome size variation in the hybrid. Notwithstanding, ribosomal DNA clusters and some DNA transposons deviated from the expectancy, showing both under and overrepresentation based on the deviation scores obtained (Figure 3C).

### 2.6. Chloroplast Reconstruction and Network Analysis

**Figure 4:**
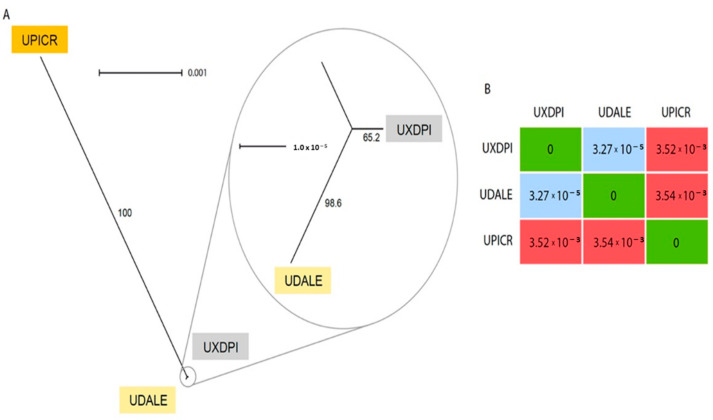
(**A**) Neighbor-Net splits graph based on uncorrected p-distances of the chloroplast matrix of *U. dalechampii* (UDALE), *U. picroides* (UPICR) and *U. dalechampii* × *U. picroides* (UXDPI). Numbers in branches indicate bootstrap support. (**B**) Distance matrix of the three taxa included in the analysis.

The chloroplast reconstruction resulted in three complete chloroplast sequences of 152,726 bp (*U. dalechampii*), 152,527 bp (*U. picroides*) and 152,746 bp (*U. dalechampii* × *U. picroides*; Supplementary Figure S1). The alignment of the three full chloroplast sequences revealed only nine variants between the hybrid and *U. dalechampii*, while *U. picroides* and the hybrid had 637 variants (the parental donors *U. dalechampii* and *U. picroides* showed 644 variants). A graphical reconstruction of the SplitsTree network is depicted in Figure 4A, including the resulting distance matrix (Figure 4B).

## 3. Discussion

### 3.1. A Hybrid with a Very Distinctive Involucral Bract Indumentum

We primarily identified putative hybrids based on morphology. At first, we thought they could be small-sized individuals of *U. dalechampii*, but after closer inspection of their capitula, we discarded such hypothesis. Indeed, these individuals presented a very distinctive involucral bract indumentum, not as softly hairy than in *U. dalechampii* nor so strongly hispid and spinulose than in *U. picroides* (Figure 1B–G), which led us to hypothesise their likely hybrid origin. All putative hybrids identified based on intermediate indumentum features –and only them– also displayed intermediate genome size values (Figure 2D), confirming the usefulness of this morphological trait to detect hybrids. It should be noted that hybrids show characteristics very close to the average between the parental species for most traits irrespective of their nature (i.e., morphological, karyological, cytogenetic or genomic) indicative either of F1 generation or subsequent generations in a context where backcrosses with parental species are excluded. Given that none of the hybrid individuals we studied produced viable seeds, we are therefore more inclined to believe they are likely F1 hybrids.

To our knowledge, the only record of hybridisation in this genus prior to our study referred to two different types of hybrids in Morocco [1,5]: one similar to *U. dalechampii* and sterile, and the other morphologically closer to *U. picroides*, although larger than both parental species, perennial, and mostly –although not completely– sterile. This second type also has a chromosome number identical to *U. picroides* (2n = 10) and a secondary metabolite spectrum similar to this species, but a distinctive essential oil profile. The hybrid we described in the present study clearly corresponds to the first type, which, according to our results, is homoploid (Figure 2) and has *U. dalechampii* as a maternal progenitor (see below). Our efforts to find individuals corresponding to the second type of hybrids in the area of study were unsuccessful. From the description provided [1,5], this hybrid type could represent a reciprocal cross where *U. picroides* acts as the maternal progenitor.

### 3.2. Genome Size Provides Support for Homoploid Hybridisation in Urospermum

Assuming a hypothetical scenario where two closely related species differing in genome size hybridise, F1 generation resulting from their cross could be expected to display genome sizes corresponding to the mean of their parental donors. Identification of homoploid hybrids can be difficult if the chromosome number and genome size of progenitors are similar [8]. Furthermore, in nature, an additional level of complexity is frequently brought by the fact that it is challenging to identify and confirm primary crosses freed from introgression, but this can be tested under glasshouse conditions in synthetic hybrids. For example, in *Hieracium* L., the genome size of F1 synthetic homoploid hybrids between *H. intybaceum* Jacq. and *H. prenanthoides* Vill. fits such assumption, with genome size values that correspond to the mean genome sizes of their progenitors [21]. Similarly, in the waterlily genus *Victoria* Lindl., the horticulturally cultivated *V*. ‘Longwood hybrid’, resulting from the cross of *V. amazonica* (Poepp.) Klotzsch and *V. cruziana* A.D.Orb., also displays both nuclear DNA content and chromosome number intermediate to the parental species [29]. As mentioned earlier, spontaneous hybridisation in the wild can be subjected to recurrent introgression once hybrids overcome fertility barriers and become compatible with progenitors, which would result in a more complex signature of genome sizes after generations (e.g., *Centaurea* [10]). Also, genomic reorganisation in homoploid hybrids could lead to deviations in genome size, such as in *Helianthus*, where hybrid taxa display larger genomes compared to their progenitors [30], and such increase could have been favoured by selection and adaptation to new ecological conditions. Our flow cytometry results, however, provide robust support for the homoploid hybrid hypothesis in *Urospermum* (Figure 2D, Table 2), since an expected hybrid with a 50% genome dosage of each parent would have a genome size of 1.88 pg/2C, and the actual value obtained for the suspected hybrid is 1.89 pg/2C. Furthermore, the intermediate chromosome count (2n = 12) obtained in the hybrid aligns with that expectation and confirms without doubts our hypothesis. So far, the hybrid specimens analysed did not produce viable seeds, which is likely to be indicative of F1 generation, but further monitoring of the areas of contact between both species will be crucial for evaluation of hybridisation dynamics between these two species.

### 3.3. Chloroplast Analysis Provides Insights into the Parentage Origin of the Hybrid

Low-coverage genome skimming approaches enable recovery of organellar DNA, including both mitochondrion and chloroplast [31], and hence their assemblies can be done directly from genomic DNA without the need for specific enrichment. Our chloroplast reconstruction provided robust support for the assessment of the origin of the hybrid taxon based on the number of SNPs shared with the hybrid and each progenitor (Figure 4). Certainly, the significant lower number of variants detected between *U. dalechampii* and the hybrid compared to *U. picroides*, strongly suggests that *U. dalechampii* acted as the maternal parent in this cross (Figure 4). These results, however, are based on sequencing data for one hybrid taxon, and do not preclude from other reciprocal spontaneous crosses that could eventually happen in natural populations where both species coexist. Nonetheless, during field observations, we noticed that *Urospermum* capitula open only a few hours per day, those of *U. picroides* usually earlier than those of *U. dalechampii*, with a short overlap when capitula of both species are open offering a window for interspecific pollen transfer. The fact that capitula of *U. picroides* open earlier could favour its role as paternal progenitor, since pollinating insects may have already visited its capitula –and collected pollen– when those of *U. dalechampii* open. Mating system could provide an additional explanation for the observed directional hybridisation, that fits the expectation of asymmetric reproductive barrier strength and gene flow from selfers (*U. picroides* [5]) to outcrossers (as is probably *U. dalechampii*, which meets the outcrossing syndrome with its capitula of significantly larger size and more florets) [32].

### 3.4. Analysis of Repetitive DNA in Urospermum and Consequences of Hybridisation in the Genome Organisation of Hybrid Taxa

As for the chloroplast reconstruction, because of the unbiassed scanning nature of genome skimming techniques, a representation of the repetitive genome can be obtained, characterised and quantified upon short-read assembly [25]. Across land plants, the repetitive DNA contribution to the genome is highly variable, ranging from around 9 to 80%, and it is heavily influenced by the ultimate size of the genome, especially in relatively small genomes (of up to 10 Gbp, [33]). The *Urospermum* taxa analysed possess small genomes, and the total amounts of repetitive DNA recovered using RepeatExplorer fall within the range described above (c. 74.16% in *U. dalechampii* and 69.74 to nearly 70.80% in *U. picroides* and the putative hybrid, respectively). Individual clustering analyses resulted in larger proportions of transposable elements recovered in *U. dalechampii* than in *U. picroides*, which were more apparent across the Ty3/Gypsy elements detected, but also noticeable for Ty1/Copia (Table 4). A similar pattern was recovered in the comparative analysis, which is based in shared DNA repeats (Figure 3B). This is something one could expect bearing in mind the larger genome of *U. dalechampii*, which is 31% larger than that of *U. picroides*. However, the proportion of tandem repeats (i.e., satellite DNA) revealed a contrasting pattern, being present in an over two-fold proportion in *U. picroides* compared to *U. dalechampii* (15.638% vs. 6.942%, Figure 3B for specific clusters). Copy numbers of satellite DNA can vary rapidly due to expansions and contractions of their arrays, which are frequently correlated to changes in genome size [22,34], but do not necessarily drive their size (e.g., *Prospero* Salisb., [35]). In our case, the larger proportions of satellite DNA in *U. picroides* seem to be counterbalanced by an overall higher incidence of most classes of dispersed elements (including both LTR and DNA transposons) contributing significantly to the larger genome of *U. dalechampii*.

Besides the influence of genome size in shaping the repetitive landscape, the slightly smaller proportion of repetitive elements observed in the hybrid given the size of its genome, could be linked to the resulting admixture and recombination processes inherent to the hybridisation process itself [36]. In general, the proportions of the main types of repetitive elements in the hybrid taxon were close to the expected values assuming a proportional genome dosage from its progenitors (Figure 3D, Table 2), but slightly larger deviations from expectancy were mostly observed clusters with a relatively small contribution to the genome (Figure 3C). Such pattern falls within the expected results of a recently formed hybrids with no genome additivity from the parental genomes [21], or where amplification of specific elements is counterbalanced by elimination of others. In plants, however, as indicated in Section 3.2, examples of genomic expansions during homoploid hybridisation can be found in other sunflower genera (e.g., *Helianthus*), where hybrids underwent genome upsizing driven by the activation of several Ty3/Gypsy retroelements [37]. An exception to the general pattern was found in the number of clusters recovered classified as ribosomal DNA, whose proportions were, in general, lower than expected in the hybrid (Figure 3C). Larger deviations of ribosomal and satellite DNA over transposable elements were already reported in hybrids of *Hieracium* and *Melampodium* L. [21,23], though in several of these examples polyploidy was also involved. Likewise, partial loss of ribosomal DNA repeats in hybrids has been reported in the past, but most evidence is based on allopolyploids, and associated to diploidisation of the genome, especially in older allopolyploids [38]. The ultimate reasons and underlying mechanisms behind the loss of ribosomal DNA are not yet completely understood, but unbalanced parent homogenisation of copies after hybridisation and polyploidisation has been reported in the past [39,40]. Also, ectopic recombination between homologous copies of repetitive DNA sequences could result in the loss of copies, and indeed, it has been seen as driving force for ribosomal repatterning in plants and insects [41,42].

## 4. Materials and Methods

### 4.1. Plant Sampling

Studied populations of *Urospermum* [*U. dalechampii*, *U. picroides* and the resulting hybrids] were growing wild in the Botanical Garden of Barcelona and nearby meadows in Montjuïc Park (Barcelona, Spain). In total, 28 individuals were sampled for morphological characterisation and flow cytometry measurements: 10 individuals of each parental species and eight of putative hybrids. Efforts have been made to sample individuals covering the morphological diversity of the species, in particular for *U. picroides*, which shows great variability. Capitula collected for morphological characterisation were either the capitulum ending the primary shoot (T), or, in few instances, the capitulum ending the distal-most lateral flowering axes (A and B; branching hierarchy letter sequence as in Wreath et al. [43]). Herbarium vouchers are deposited in herbarium BC (Botanical Institute of Barcelona).

### 4.2. Chromosome Counts

Roots were collected from cultivated plants of *U. dalechampii*, *U. picroides* and *U. dalechampii* × *U. picroides* in pots at the glasshouse facilities of the Botanical Garden of Barcelona (Accessions OH 670, OH 653 and OH 660 respectively). These three accessions were also used for genome size analyses (among others) and Illumina sequencing. Briefly, young and healthy roots were pre-treated in 0.05% aqueous colchicine at RT for 3 h, fixed in fresh absolute ethanol and glacial acetic acid (3:1) for 2–5 h at RT, and stored in the fixative at −20 °C. Roots were rinsed in distilled water for 10 min, hydrolysed in 1N HCl for 5 min at 60 °C, stained in 1% aqueous aceto-orcein for 2 h, and finally, the excised root tip was squashed in a drop of 45% acetic acid-glycerol (9:1). Preparations were observed with a Zeiss Axioplan microscope, and the best metaphase plates were photographed with a Zeiss AxioCam HRm camera. Images were analysed with Axio Vision Ac software version 4.2.

### 4.3. Flow Cytometry Measurements

Genome size was determined using propidium iodide flow cytometry with a CyFlow Space instrument (Sysmex-Partec, Norderstedt, Germany), fitted with a 100-mW green solid-state laser (Cobolt Samba) following the one-step procedure [44] with modifications as described in [45]. We used *Petroselinum crispum* (Mill.) Fuss. ‘Champion Moss Curled’ [46] as calibration standard, and the Cystain Ox Protect and PI Absolute buffers (Sysmex-Partec, Germany). Sample preparation was made according to the manufacturer’s instructions. For each accession analysed, one sample was prepared, and assessed three times on the flow cytometer. The nuclear DNA content of each sample run was estimated by recording at least 1000 nuclei per fluorescence peak. Resulting output histograms were analysed using the FlowMax software (v. 2.9, Sysmex-Partec GmbH) for statistical calculations.

### 4.4. Statistical Analyses

Data visualisation and analyses were carried out with the package ggplot2 [47] in R v.3.2.2 [48]. Principal components analysis (PCA) of log-transformed capitula measurements was performed using the ‘prcomp’ function on a dataset including five variables that describe capitulum shape and size (capitulum height, capitulum width, involucre height, involucre width and receptacle width). A correlation analysis was performed to ensure that variables presented correlation coefficient values < 0.90. After verifying assumptions of homogeneity of variances (with Bartlett’s test, ‘bartlett.test’ function) and of normality on residuals (with Shapiro test, ‘shapiro.test’ function, and QQ-plot), we proceeded to a one-way ANOVA and performed multiple pairwise comparisons between the means of groups (Tukey honest significant differences, ‘TukeyHSD’ function). When the hypothesis of homogeneity of variance and/or normality of residuals was rejected, we performed a Krustal-Wallis test (‘krustal.test’ function) and calculated pairwise comparisons between groups using the pairwise Wilcoxon rank sum test (‘pairwise.wilcox.test’ function) with Bonferroni correction.

### 4.5. DNA Isolation and Next Generation Sequencing

The three accessions selected for sequencing represent each parental progenitor (i.e., *U. dalechampii* and *U. picroides*), and interspecific hybrid (*U. dalechampii* × *U. picroides*). For each accession, about 10–30 mg of silica-dried leaf tissue was homogenised using a Mixer Mill MM 301 (Retch, Haan, Germany). Genomic DNA extraction was carried out using the E.Z.N.A. SP Plant DNA Kit (Omega Bio-Tek, Norcross, GA, USA) following the manufacturer’s protocol. The quantity of the extracted DNA was measured with a Qubit 3.0 Fluorometer (Thermo Scientific, Waltham, MA, USA). Paired-end shotgun libraries (TruSeq DNA PCR-free, 2 × 150 bp) with an average insert size of 450 bp were prepared and sequenced by Macrogen Inc. (Seoul, Korea) on a NovaSeq 6000 Illumina sequencing system (Illumina, San Diego, CA, USA). The quality of sequencing data was assessed using FastQC (http://www.bioinformatics.babraham.ac.uk/projects/fastqc/ downloaded on the 23 September 2021) and reads were pre-processed using the FASTX-Toolkit (http://hannonlab.cshl.edu/fastx_toolkit/ downloaded on the 23 September 2021) and Trimmomatic v.0.39 (http://www.usadellab.org/cms/?page=trimmomatic downloaded on the 23 September 2021) [49] with options AVGQUAL:20 MINLEN:110 LEADING:20 TRAILING:20 SLIDINGWINDOW:4:20.

### 4.6. Graph-Based Clustering in RepeatExplorer 2 and Transposable Element Annotation

Analysis and identification of DNA repeats was carried out using the RepeatExplorer 2 pipeline (https://repeatexplorer-elixir.cerit-sc.cz/galaxy/ accessed on the 1 October 2021), a GALAXY-based server for characterisation of repetitive elements based on similarity clustering of Illumina paired-end reads [24,50]. Pre-processed paired FASTQ reads were converted to FASTA format, interlaced and trimmed to 100 bp prior to the clustering analysis with FASTX-Toolkit. A preliminary round of clustering was performed with the original datasets [*U. dalechampii* = 19,848,388 reads, *U. picroides* = 16,219,976 reads, and *U. dalechampii* × *U. picroides* = 19,837,944 reads] to determine the maximum number of reads for each species to include representative of the same genomic proportions. This analysis was carried out using the default settings (90% similarity over 55% of the read length, and cluster size threshold = 0.01%). Each set of reads was randomly down-sampled according to their genome size to represent reads comprising 20% of the genome of each species (i.e., genome proportion = 0.20×, [*U. dalechampii* = 2,240,000 reads, *U. picroides* = 1,595,714 reads and *U. dalechampii* × *U. picroides* = 1,916,000). Note that after clustering analysis, organelle reads were excluded, and so final genome proportions analysed resulted of 0.19–0.20×. Automated repeat classification was based on connection-based clustering via paired-end reads and BLAST(n, x) similarity searches to REXdb [51], a comprehensive database of conserved protein domains in retrotransposons. Output directories were individually examined for a final manual annotation based on protein domain hits and quantification of clusters and connections to superclusters. TAREAN [52] and Tandem Repeat Finder [53] were used for the discovery of potential tandem repeats (e.g., satellites). Besides the individual clustering, a comparative clustering analysis was carried out using a combined dataset of 2,831,000 reads (each species at a genome proportion of c. 0. 10×; i.e., *U. dalechampii* = 1,120,000 reads, *U. picroides* = 753,000 reads and *U. dalechampii* × *U. picroides* = 958,000). A four-letter prefix identity code was added to each sample dataset and used as the input to Repeat Explorer as described above. Repeat annotation of shared clusters between the two species was done following the same parameters as for the individual analyses. Cluster abundances were analysed by comparing their absolute sizes (in read number). Baseline statistics including, genome proportion (in percentage) and abundance (Mb/1C) of DNA repeats identified were calculated. Based on the number of reads observed in the hybrid, we calculated the expected number of reads in each parent considering the genome size of each parent and the hybrid, applying the following formula: Reads__P_ = (GS__P_ × No. Reads__HYB_)/GS__HYB_. Deviation scores for clusters were calculated dividing the observed cluster size by the expected cluster sizes, as in Zagorski et al. [21].

### 4.7. Assembly of Chloroplast Genomes and Network Analysis

Raw reads were assembled using NOVOPlasty v.4.3.1 [54] using the default parameters, which resulted in three contigs for each species analysed. The resulting contigs were mapped to a reference plastome of *Hypochaeris radicata* L. (Genbank Acc. MH746729) using Geneious Prime 2021.2.2 (https://www.geneious.com downloaded on the 11 October 2021). All contigs used covered 100% of the reference, and therefore, the consensus sequence from the mapping was extracted. To corroborate the veracity of the analysis, raw reads were further cleaned and paired with Trimmomatic v.0.39 [49] as indicated above. Cleaned reads were mapped to the consensus sequence to evaluate if there were discrepancies in any position, using a similarity threshold of 90%. No variants were found in any of the three assembled chloroplasts and they were further annotated with OrganellarGenomeDRAW v.1.3.1. [55]. Finally, the three chloroplast sequences were aligned using MAFFT v.7.450 [56] with standard parameters and manually adjusted. Variations (i.e., including insertions, gaps and SNPSs) between individuals were found with the ‘Find Variations/SNPs’ option in Geneious Prime. The nexus alignment file was used to conduct a distance network analysis in SplitsTree v.4.17.1 [57] under a Neighbour-Net approach [58], with a bootstrap of 1000 runs obtained with several distance settings without any change (Uncorrected_P, JukesCantor, K2P and HKY85).

## 5. Conclusions

We have shown in this study that, besides the genomic consequences of hybridisation reported here, *Urospermum* hybrids can be easily identified in the field based on morphological traits (i.e., involucre characters). We believe that, if they had been present for long time in areas where both parent species co-exist (in addition to the record of Morocco), they would have been reported in botanical literature, which is not the case. For that reason, hybridisation between these taxa is likely a recent process. It resulted from interspecific pollen transfer, which could have been triggered by factors promoting the co-occurrence and synchronisation of flowering behaviour of *Urospermum* species, such as changes in their distribution and/or phenology. Increasing local abundance of *U. dalechampii* and *U. picroides* have been reported in Montjuïc in recent years (Samuel Pyke, personal communication). For that reason, future studies will be needed to monitor the extent of hybridisation beyond the current area of study, as well as to evaluate the impact on the biology of the genus. There is indeed, growing evidence on the effect of global change in increasing hybridisation opportunities by altering reproductive isolation barriers [59], and this could be especially relevant if any of these hybrids overcomes fertility issues and becomes established.

## Figures and Tables

**Table 1:** Morphological data for *Urospermum dalechampii*, *U. dalechampii* × *U. picroides* and *U. picroides* at Montjuïc (Catalonia, Spain).

Traits	*U. dalechampii*		*U. dalechampii* × *U. picroides*		*U. picroides*	
	Mean (SD ^1^)	Min–Max	Mean (SD ^1^)	Min–Max	Mean (SD ^1^)	Min–Max
Plant height ^2,3^	288.5 (116.60)	125–485	182.10 (74.80)	65–278	291.42 (224.74)	30–800
N capitula ^4^	1.2 (0.42)	1–2	1.6 (0.84)	1–3	6 (6.52)	1–20
Receptacle width ^2^	10.43 (2.06)	7.78–14.89	6.08 (1.26)	4.59–7.87	5.78 (2.73)	1.37–8.62
N Florets ^5^	207 (34.66)	163–267	89.2 (33.9)	36–140	83.08 (45.53)	18–139
Capitulum width ^2^	55.76 (9.11)	42.09–68.90	32.81 (5.54)	24.57–41.22	21.84 (4.61)	13.68–28.35
Capitulum height ^2^	30.89 (3.54)	24.76–36.51	22.65 (2.39)	19.47–27.54	22.21 (3.90)	13.88–27.09
Involucre width ^2,4^	24.46 (3.65)	18.33–29.28	20.82 (5.02)	11.73–26.85	12.74 (3.92)	5.57–16.6
Involucre height ^2^	18.72 (1.37)	16.31–2086	16.26 (2.31)	12.72–20.42	17.99 (3.43)	11.7–21.91

^1^ SD: standard deviation. ^2^ Measurements in mm. ^3^ Vertical distance from the ground to the highest point of the plant. ^4^ Number of capitula per flowering shoot. ^5^ Number of florets per capitulum.

**Table 2:** Genome size data for *Urospermum dalechampii*, *U. dalechampii* × *U. picroides* and *U. picroides* at Montjuïc (Catalonia, Spain).

*Urospermum* Species	2C (pg) ± SD ^1^	N ^2^	CV_plt_^3^	CV_std_^4^	1C (pg)	1C (Mbp) ^5^
*U. dalechampii*	2.26 ± 0.01	10	3.99	2.59	1.13	1105.14
*U. dalechampii* × *U. picroides*	1.89 ± 0.01	8	3.88	3.00	0.95	924.21
*U. picroides*	1.51 ± 0.01	10	3.67	2.71	0.76	738.39

^1^ SD: standard deviation. ^2^ N: number of individuals measured. ^3^ CV_plt_: coefficient of variation for *Urospermum* accessions (in %). ^4^ CV_std_: coefficient of variation for the calibration standard (in %). ^5^ 1 pg = 978 Mbp [28].

**Table 3:** Genome skimming details of the three *Urospermum* taxa studied.

Urospermum Species	1C/Mbp	No. of PE Reads after QC	No. of Reads Analysed	No. of Reads Analysed *	Coverage
*U. dalechampii*	1066.06	19,848,388	2,240,000	2,049,890	0.19×
*U. picroides*	733.5	16,219,976	1,585,714	1,439,699	0.19×
*U. dalechampii* × *U. picroides*	899.76	19,837,944	1,916,000	1,799,837	0.20×

* Excluding organelle reads.

**Table 4:** Repeat composition inferred in the studied *Urospermum* species and the putative hybrid *U. dalechampii* × *U. picroides*.

		GENOME PROPORTION (GP)					
		*U. dalechampii*		*U. picroides*		Hybrid	
Repeat Type	Lineage	[%]	[Mb]	[%]	[Mb]	[%]	[Mb]
Ty1/Copia		24.007	255.911	20.681	151.698	22.483	202.292
	SIRE	18.026	192.154	17.064	125.168	17.690	159.163
	Angela	2.990	31.873	2.903	21.291	2.974	26.758
	TAR	0.308	3.280	0.357	2.622	0.430	3.873
	Bianca	0.517	5.514	0.241	1.764	0.377	3.394
	Ale	0.209	2.226	0.116	0.853	0.016	0.147
	Ivana	0.309	3.294	0.000	0.000	0.028	0.250
	Tork	1.632	17.397	0.000	0.000	0.968	8.706
	Ikeros	0.016	0.174	0.000	0.000	0.000	0.000
Ty3/Gypsy		10.938	115.443	4.255	31.207	6.693	60.219
	Tekay	9.718	103.591	3.745	27.467	5.632	50.677
	Athila	0.981	9.337	0.373	2.734	0.871	7.833
	CRM	0.102	1.085	0.137	1.006	0.160	1.443
	Retand	0.118	1.256	0.000	0.000	0.030	0.266
	Reina	0.020	0.174	0.000	0.000	0.000	0.000
LTR		0.865	9.222	0.074	0.545	1.849	16.639
Other repeats							
	Pararetrovirus	0.874	9.316	0.188	1.378	0.645	5.803
	LINE	0.720	7.678	0.012	0.087	0.406	3.650
DNA transposons		3.016	32.999	1.168	8.571	1.337	12.029
	TIR/Enspm-CACTA	0.541	5.765	0.000	0.000	0.190	1.713
	TIR/MuDR-Mutator	0.975	11.439	0.766	5.619	0.416	3.742
	TIR/haT	0.349	3.721	0.000	0.000	0.153	1.380
	TIR/PIF-Harbinger	0.082	0.666	0.050	0.366	0.083	0.748
	TIR/Mariner	0.658	7.018	0.000	0.000	0.000	0.000
	Helitron	0.412	4.390	0.353	2.586	0.494	4.445
Tandem repeats							
	rDNA	4.173	44.489	4.141	30.374	2.954	26.583
	Satellite	6.942	74.004	15.638	114.703	11.657	104.884
Unclassified		4.486	48.151	4.822	35.368	4.834	43.498
GP < 0.01%		18.142	193.390	18.765	137.645	17.934	161.367
Total repeats		74.164	790.602	69.744	511.575	70.793	636.964
Single copy		25.836	275.415	30.256	221.926	29.207	262.796

## Data Availability

The datasets presented in this study can be found in online repositories. The names of the repository and accession number(s) can be found below: https://www.ncbi.nlm.nih.gov/. Bioproject PRJNA787421: SAMN23802836, SAMN23802837, SAMN23802838.

## Supplementary Materials

The following are available online at https://www.mdpi.com/article/10.3390/plants11020182/s1, Supplementary Table S1 and Supplementary Figure S1.
