# Supplementary material for: Morphological and Genome-Wide Evidence of Homoploid Hybridisation in Urospermum (Asteraceae)
Source: Plants (Basel). 2022 Jan 11;11(2):182. doi: 10.3390/plants11020182 (PMC8779322; doi:10.3390/plants11020182)
Supplement: Supplementary file 1 [file plants-11-00182-s001.zip › plants-1531646-supplementary_figure_1.pdf]

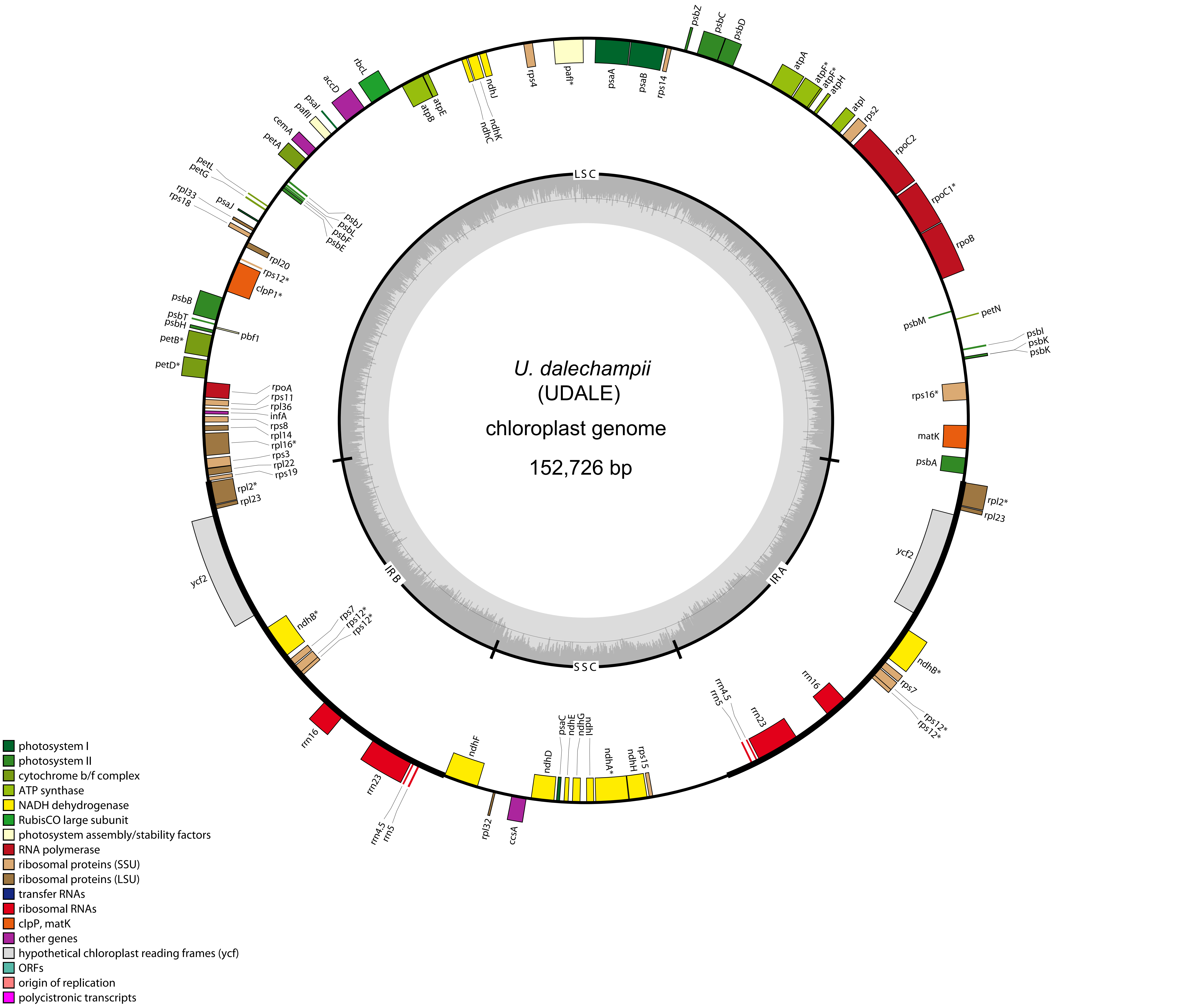

U. dalechampii
(UDALE)
chloroplast genome
152,726 bp
LSC
SSC
IRA
IRB
psbZ
psbC
psbD
atpA
atpF*
atpH
atpI
rps2
rpoC2
rpoC1*
rpoB
petN
psbM
psbI
psbK
rps16*
matK
psbA
rpl2*
rpl23
ycf2
ndhB*
rps7
rps12*
rrn16
rrn23
rrn4.5
rrn5
rps15
ndhH
ndhA*
ndhI
ndhG
ndhE
psaC
ndhD
ccsA
rpl32
ndhF
rpl2*
rpl23
rps19
rpl22
rps3
rpl16*
rpl14
rps8
infA
rpl36
rps11
rpoA
petD*
petB*
psbH
psbT
pbf1
psbB
clpP1*
rps12*
rpl20
rps18
rpl33
psaJ
petG
petL
psbJ
psbL
psbF
psbE
petA
cemA
pafII
psaI
accD
rbcL
atpB
atpE
ndhC
ndhK
ndhJ
rps4
pafI*
psaA
psaB
rps14
photosystem I
photosystem II
cytochrome b/f complex
ATP synthase
NADH dehydrogenase
RubisCO large subunit
photosystem assembly/stability factors
RNA polymerase
ribosomal proteins (SSU)
ribosomal proteins (LSU)
transfer RNAs
ribosomal RNAs
clpP, matK
other genes
hypothetical chloroplast reading frames (ycf)
ORFs
origin of replication
polycistronic transcripts

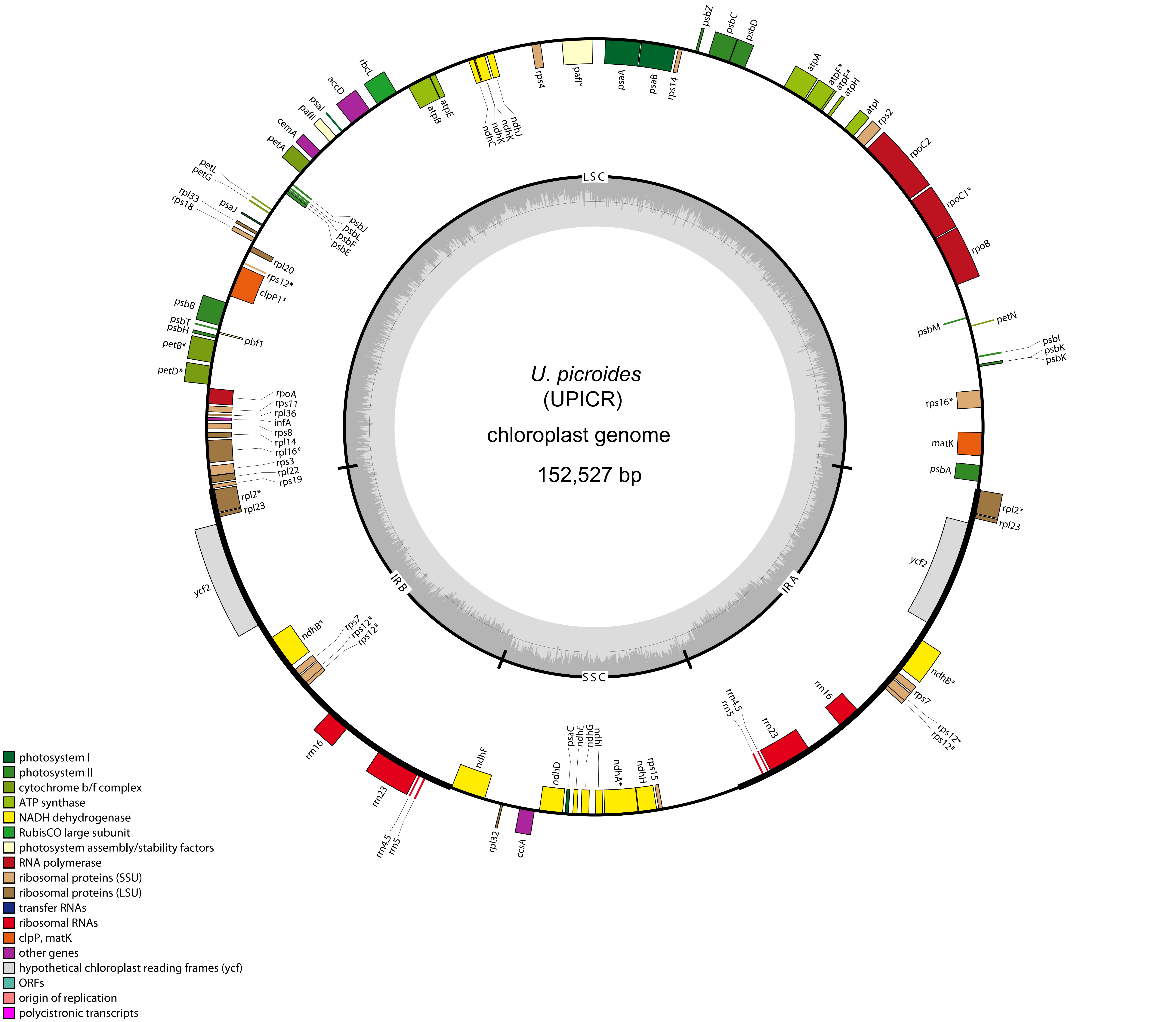

U. picroides
(UPICR)
chloroplast genome
152,527 bp
LSC
SSC
IRA
IRB
psbZ
psbC
psbD
atpA
atpF*
atpF*
atpH
atpI
rps2
rpoC2
rpoC1*
rpoB
petN
psbM
psbI
psbK
psbK
rps16*
matK
psbA
rpl2*
rpl23
ycf2
ndhB*
rps7
rps12*
rps12*
rrn16
rrn23
rrn4.5
rrn5
rps15
ndhH
ndhA*
ndhI
ndhG
ndhE
psaC
ndhD
ccsA
rpl32
ndhF
rrn23
rrn4.5
rrn5
rrn16
ndhB*
rps7
rps12*
rps12*
ycf2
rpl2*
rpl23
rpoA
rps11
rpl36
infA
rps8
rpl14
rpl16*
rps3
rpl22
rps19
petD*
petB*
psbT
psbH
pbf1
psbB
clpP1*
rps12*
rpl20
rpl33
rps18
psaJ
petL
petG
psbJ
psbL
psbF
psbE
petA
cemA
pafII
psaI
accD
rbcL
atpB
atpE
ndhC
ndhK
ndhJ
rps4
pafI*
psaA
psaB
rps14
photosystem I
photosystem II
cytochrome b/f complex
ATP synthase
NADH dehydrogenase
RubisCO large subunit
photosystem assembly/stability factors
RNA polymerase
ribosomal proteins (SSU)
ribosomal proteins (LSU)
transfer RNAs
ribosomal RNAs
clpP, matK
other genes
hypothetical chloroplast reading frames (ycf)
ORFs
origin of replication
polycistronic transcripts

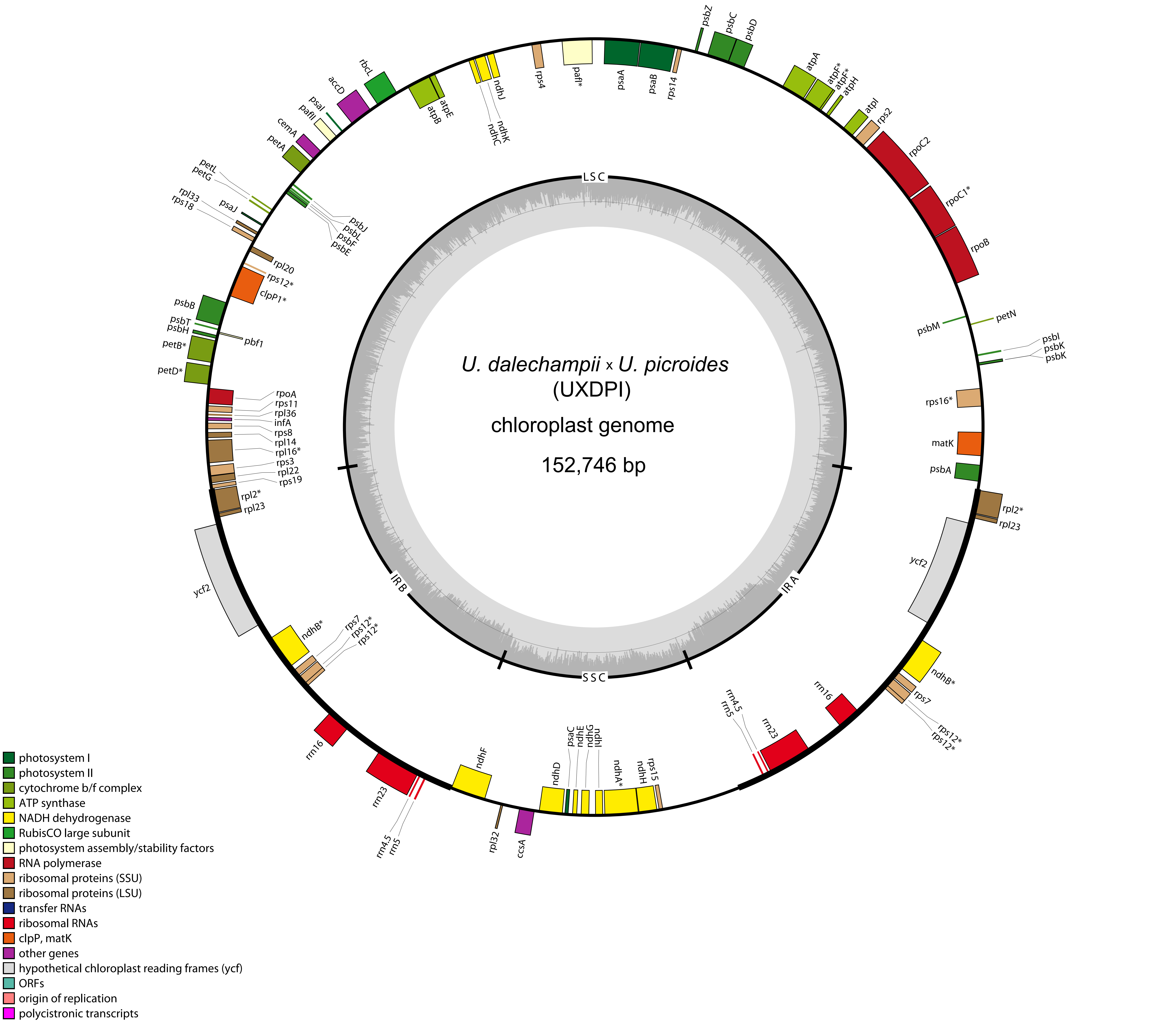

U. dalechampii × U. picroides
(UXDPI)
chloroplast genome
152,746 bp
LSC
SSC
IRA
IRB
psbZ
psbC
psbD
atpA
atpF*
atpF*
atpH
atpI
rps2
rpoC2
rpoC1*
rpoB
petN
psbM
psbI
psbK
psbK
rps16*
matK
psbA
rpl2*
rpl23
ycf2
ndhB*
rps7
rps12*
rps12*
rrn16
rrn23
rrn4.5
rrn5
ndhD
psaC
ndhE
ndhG
ndhI
ndhA*
ndhH
rps15
ccsA
rpl32
ndhF
rps4
pafI*
psaA
psaB
rps14
ndhJ
ndhK
ndhC
atpE
atpB
rbcL
accD
psaI
pafII
cemA
petA
petL
petG
psbJ
psbL
psbF
psbE
rpl33
rps18
psaJ
rpl20
rps12*
clpP1*
psbB
psbT
psbH
pbf1
petB*
petD*
rpoA
rps11
rpl36
infA
rps8
rpl14
rpl16*
rps3
rpl22
rps19
photosystem I
photosystem II
cytochrome b/f complex
ATP synthase
NADH dehydrogenase
RubisCO large subunit
photosystem assembly/stability factors
RNA polymerase
ribosomal proteins (SSU)
ribosomal proteins (LSU)
transfer RNAs
ribosomal RNAs
clpP, matK
other genes
hypothetical chloroplast reading frames (ycf)
ORFs
origin of replication
polycistronic transcripts
